# “I Just Glove up and Do What Has to Be Done”: A Mixed-Methods Exploration of Dementia Care Challenges and Care Management Strategies

**DOI:** 10.3390/ijerph23020254

**Published:** 2026-02-17

**Authors:** Amanda N. Leggett, Natasha Nemmers, Sophia Tsuker, Laura N. Gitlin, Helen C. Kales

**Affiliations:** 1 Institute of Gerontology, Wayne State University, Detroit, MI 48202, USA; leggetta@wayne.edu (A.N.L.);; 2 College of Nursing and Health Professions, Drexel University, 3141 Chestnut St., Philadelphia, PA 19104, USA; 3 Department of Psychiatry and Behavioral Sciences, University of California Davis, Davis, CA 95616, USA

**Keywords:** dementia caregiving, dementia management strategies, behavioral and psychological symptoms of dementia, memory, activities of daily living

## Abstract

**Highlights:**

**Public health relevance—How does this work relate to a public health issue?**

- Family care partners adopt a variety of care management strategies to navigate care for persons living with dementia (PLwD), often without formal training.
- Behavioral and psychological symptoms of dementia (BPSD) are associated with burden, institutionalization, and mortality, making effective management strategies critical.

**Public health significance—Why is this work of significance to public health?**

- BPSD, particularly agitation, were the most challenging and consistently stressful dementia symptoms reported by care partners.
- Care partners used active management for functional and cognitive care, but BPSD coincided with more critical, rigid, and disengaging strategies that reduce well-being for both care partners and PLwD.

**Public health implications—What are the key implications or messages for practitioners, policy makers and/or researchers in public health?**

- Future interventions should help care partners practice more adaptive behavioral management rather than punitive strategies like criticism.
- Tailored interventions targeting care partners’ specific management styles and unique care stressors may improve care provision and mental health.

**Abstract:**

Family care partners for persons living with dementia (PLwD) adopt a variety of care management strategies to navigate care. We utilize a convergent parallel mixed-methods design to integrate family care partners’ descriptions of care challenges and associated management approaches. Primary family care partners for PLwD (n = 100) were interviewed about their management of a care challenge (qualitative), the PLwD’s function, behavior and cognition (quantitative), and their management strategies (qualitative/quantitative). Care challenges and strategies were compared across qualitative content analysis and t-tests. Care partners providing more functional (r = 0.34, *p* < 0.01) and cognitive (r = 0.30, *p* < 0.01) care used more active management strategies, whereas care partners providing greater behavioral care (r = 0.34, *p* < 0.001) reported more criticism, with similar themes seen qualitatively. Active management may be required when dementia severity increases, yet criticism may emerge with greater behavioral severity. Because criticism is associated with more negative outcomes, future interventions should help care partners practice more adaptive behavioral management.

## 1. Introduction

Dementia, as a clinical syndrome, presents with numerous symptoms that pose challenges with which family care partners, such as spouses and adult children, must cope with and manage. Behavioral and psychological symptoms of dementia (BPSD), for example, are associated with a host of negative care outcomes including depression, burden, morbidity, institutionalization, and even early mortality among persons living with dementia (PLwDs) [1,2,3,4]. While symptoms stem from neurodegeneration, their severity and frequency may be influenced by factors at multiple levels: PLwD (e.g., pain), care partner (e.g., mismatch of expectations and dementia severity), and the environment (e.g., overstimulation) [3].

Existing theoretical models in family caregiving research posit that these symptoms are a key part of the stress process for care partners. For example, Stress Process Models suggest that primary stressors (e.g., memory loss, BPSD, and activities of daily living [ADL]) and the care partner’s perceptions of these stressors are key predictors of both care partner and PLwD outcomes (e.g., burden, institutionalization) [2,5,6,7]. Similarly, stress is experienced when an individual’s resources are perceived to be inadequate in meeting the stressor [8]. Thus, in dementia care, coping and care management are dynamic cognitive–behavioral processes aimed at mitigating and controlling the impact of the stressors care partners face. Such strategies used by care partners may vary based on the nature of the stressor and whether the care partner appraises that stressor as within their control [8].

Most family care partners enter their care role as a familial responsibility with no prior training or time to prepare; this lack of readiness can result in a diverse array of adopted care management strategies [9,10]. Just how care partners manage these symptoms, however, is rarely explored in the literature. Yet this exploration is critical because punitive care strategies (e.g., criticism, yelling, confrontation) may create negative feedback loops that exacerbate symptoms or lead to potentially harmful behavior within the care partner–PLwD dyad [11]. Punitive strategies such as criticism are used regularly by approximately 15% of care partners [12]. On the other hand, research on strength-based approaches to caregiving also emphasizes how care partners mobilize their individual strengths in care provision [13]. Care partners adopt a number of adaptive care management strategies, such as diversion, teamwork, affection, emotional support, encouragement, and environmental modifications [11,14,15]. Further, while behavioral responses to dementia symptoms can be modified and validated behavioral interventions exist, recent meta-analytic evidence shows only a small effect size for such interventions on care partner outcomes [16]. The aim of the current study was to use a convergent parallel mixed-methods design to identify dementia care partner’s behavioral management approaches towards specific symptoms of dementia and contrast these approaches by categories of symptoms [17]. Understanding the types of strategies care partners use to manage challenging symptoms of dementia can help us identify how we can better individualize behavioral care recommendations, improve care for the PLwDs, and reduce care stress.

### 1.1. Dementia Care Management

Symptoms of dementia present a challenge to care partners for a variety of reasons: they counter social norms, reflect a declining personhood of the PLwD, and represent a change in the nature of the relationship between PLwD and care partner [18]. The three most common categories of dementia symptoms studied in relation to care partner stress are cognitive impairment, functional impairment (ADL and IADL deficits), and the presence of BPSD [19]. Fauth and Gibbons found that while memory was the most frequently reported care problem, it was the least distressing to care partners [20]. Among BPSD specifically, apathy, depression, appetite, and agitation were most frequent, while delusions, agitation, and irritability were the most distressing to care partners [20]. However, regarding how care partners manage such symptoms, prior work has primarily focused on care partner’s management of BPSD or a specific IADL like medication management [21].

### 1.2. Qualitative Studies

Small qualitative studies exploring management of BPSD suggest care partners use strategies including administering psychotropic medication, attempting to understand the underlying trigger of the behavior, employing restraints or punitive actions, addressing physiological needs (e.g., pain, fatigue), adapting communication, environmental modifications, encouraging meaningful activities (e.g., exercise, chores), using social support, humor, and staying calm to dissipate the situation [13,22,23]. Further, care partners described switching between different management strategies throughout the day depending on the presenting behavioral challenge; however, which strategies were used to manage specific care challenges was not investigated [22].

### 1.3. Quantitative Studies

On the other hand, quantitative studies have been used to explore management approaches in association with specific care symptoms. For example, studying Alzheimer’s disease care partners in Brazil, Delfino and colleagues [24] considered whether certain BPSD were associated with scale scores on the dementia management strategies scale (criticism—criticizing, yelling or threatening; active management—monitoring, assisting, engaging, and other behaviors to modify the PLwD’s environment/routine; encouragement—praising, discussing emotions, and thinking positively). They found both active management and criticism to be broadly used to manage BPSD, with encouragement only associated with elation/euphoria. Similarly, de Vugt and colleagues [25] found that care partner’s lack of understanding and acceptance of the PLwD was associated with more “hyperactive” BPSD (e.g., irritability, agitation) compared to care partners who used more functional strategies such as support. Care partners who reported higher BPSD burden in their PLwDs used more disengagement (e.g., wishful thinking, withdrawing from problems), whereas engagement coping strategies (e.g., problem solving, social support) were associated with lower PLwD BPSD burden [23,26].

### 1.4. Current Study

In the current study, we use quantitative and qualitative data from the same care partners on their care management strategies related to cognitive problems, BPSD, and functional impairment (ADL/IADLs) of the PLwD. Our exploration focuses on (1) identifying the most commonly reported care challenges; (2) determining associations of specific care management strategies with care recipients’ cognitive impairment, functional impairment and BPSD; and (3) comparing and contrasting qualitative themes with quantitative reports of care management. A mixed-methods exploration of care management provides greater nuance and evidence to the limited qualitative and quantitative work on care management, allowing us to examine whether care management strategies are consistent as measured across multiple modalities. It also enables the comparison of real-world case scenarios with scale self-reported typical management behaviors. Based on previous research, we hypothesized that BPSD would be the most frequently reported care challenges and associated with greater use of criticism, whereas cognitive symptoms and ADL/IADLs would be associated with use of active management strategies.

## 2. Materials and Methods

### 2.1. Participants

Participants were family or friend adult care partners (n = 100) who held primary responsibility of care for a PLwD (age-related dementia diagnosis excluding mild cognitive impairment). Care partners of PLwDs living in an assisted care facility or with a life expectancy of less than 6 months were excluded due to the impact on management strategies.

### 2.2. Procedure

The STYLE study uses a mixed-methods design to assess care partners’ cognitive–behavioral care styles and associations with biopsychosocial care outcomes (e.g., burden, sleep). For this study, we focus on mixed-methods assessments of care management and the PLwDs’ symptoms. Interviews were ninety minutes and conducted between January and December 2018. Care partners were interviewed in their homes or a public place of their choosing within 60 miles of [blinded]. The qualitative section lasted approximately 10 min (range: 5–25 min) and asked care partners to describe a care challenge they had faced recently with the PLwD, why it was challenging, and how they responded to the challenge. The quantitative section assessed care management and symptoms of dementia using scales as described below. Signed informed consent was provided by all participants, and they received a small stipend for participation. All procedures were approved by the [blinded] IRB.

### 2.3. Measures and Analysis

This study uses a convergent parallel mixed-methods design whereby qualitative and quantitative data were collected concurrently and analyzed separately, with results being interpreted together as described below [17].

### 2.4. Care Challenges: Qualitative Coding

The principal investigator and research assistant coded care partners’ described challenges, largely consistent with the quantitative measures as described below, as primary or secondary with consensus reached by the coders. However, we also coded challenges not included in the quantitative measures. Primary care challenges were any challenge a care partner listed that resulted in a discrete cognitive and/or behavioral response. In some instances, care partners described multiple principal care challenges, and thus, the count is greater than the sample size of 100. However, care partners often named a functional or cognitive-based care challenge, yet described an underlying behavioral challenge (e.g., stated the challenge was medication management but actually described agitation or resistance to care). In addition, care partners would often list several challenges but only describe their response to one or two. In these cases, we termed named challenges without a discrete cognitive and/or behavioral response as secondary. These were described descriptively but not incorporated in the full qualitative analysis.

### 2.5. Care Challenges: Quantitative Measures

Care challenges were assessed with measures of IADLs/ADLs (the Care partner Assessment of Function and Upset; CAFU), BPSD (Neuropsychiatric Symptom Inventory-Clinician Rating Scale; NPI-C), and cognitive symptoms (Dementia Severity Rating Scale; DSRS). The CAFU [27] assesses care partners’ assistance with eight IADLs (e.g., shopping, financial management) and seven ADLs (e.g., bathing, dressing) and associated distress with providing assistance (0—not at all stressful to 4—extremely stressful). We listed IADL/ADLs by the frequency of care partners providing help with each task. Mean scores of care partner’s reported distress related to helping with the task are also reported. The NPI-C [28] assesses past month occurrence of 14 BPSDs such as apathy, aggression, and delusions, and care partner’s distress rating associated with each symptom (0—not distressing to 5—extremely distressing; mean scores reported). The DSRS [14] assesses key facets of disease impairment such as memory, personal care, and community activities. For this study, we focused on cognitive symptoms including memory, speech and language, recognition of family members, orientation to time, orientation to place, and ability to make decisions. Care partners rated the PLwD’s impairment in each area with a higher score indicating greater impairment. These items have varying severity indicators ranging from 5 to 7; thus, we divided each symptom as at or below the mid-severity level versus above the mid-severity level (thus, a severity rating of 0 to 5 would be split as 0–2 and 3–5 and a rating of 0 to 6 would be split as 0–3 and 4–6). Cognitive symptoms were organized according to the frequency of care partners providing care for a PLwD above the mid-severity level. Mean impairment scores for each symptom are also reported.

### 2.6. Care Management: Qualitative Assessment

Watkins’ “rigorous and accelerated data reduction” (RADaR) technique [29] was utilized to code and analyze qualitative text for utilized care management strategies. Based on a phenomenological qualitative approach [30], RADaR reduces and codes data specific to the research question, in our case to define care management strategies in response to the coded care challenges. Codes and definitions were iteratively refined after which the finalized focused codes were applied to each participant, with team consensus.

Due to the available frequency of each primary care challenge reported in the qualitative analyses, we describe the most prevalent care management codes for the following care challenges: ADLs, BPSD (excluding agitation), agitation, and cognitive decline which coincide with our quantitative measures. While a variety of care management strategies were utilized across the types of care challenges, describing the most frequent qualitative codes allows us to explore response patterns across categories. The most frequent codes, their definitions, and representative quotes are presented in Supplementary Table S1.

### 2.7. Care Management: Quantitative Measures

The Dementia Management Strategies (DMS) Scale [31] is a 28-item measure of the management strategies care partners utilize to deal with the PLwD’s behaviors in the last month. This scale is divided into three subscales classifying types of management strategies including the following: criticism (11 items, e.g., “I blamed my relative for having created the difficulties,” a = 0.85), active management (9 items, e.g., “I tried to divert my relative’s attention when they began to feel upset,” a = 0.65), and encouragement (8 items, e.g., “I showed special amounts of physical affection,” a = 0.72) with items on a scale of 1—never to 5—most of the time. Using t-tests, we considered whether mean scores on each subscale of the DMS—criticism, active management, and encouragement—differed by whether care partners assisted with each ADL/IADL, cared for a PLwD with each BPSD, and whether the care partner had a PLwD with above-average severity on each cognitive symptom. We further utilized Spearman correlation tests to consider associations between overall scores on the CAFU, NPI, and cognitive symptoms of the DSRS with the DMS subscales.

## 3. Results

On average, care partners were 64 years of age (SD = 16.2); 59% were spouses, 31% adult children, and 10% another relative or friend. The majority were female (74%), White (82%), and college educated (72%). Over half of the care recipients had Alzheimer’s disease (52%), 16% had dementia with Lewy Bodies, and 32% had another dementia diagnosis (e.g., vascular dementia). See Table 1.

### 3.1. Care Challenges

Frequencies of all primary and secondary care challenges are reported in Table 2, with specific challenges being reported only if they had a frequency of five or greater. The most frequently reported care challenges included BPSD (with 45% of the sample describing a BPSD), cognitive decline (18%), and activities of daily living (15%). Within BPSD reported, the most frequent challenges included agitation, delusions and perseveration, while the most common facets of cognitive decline described as challenging were executive dysfunction and memory impairment. Several challenges related to ADLs were described (e.g., bathing, dressing), though none had a frequency of 5 or greater. Of note, 10% of the sample described care coordination issues and 5% described handling the PLwD’s struggle with his/her decline and diminishing autonomy as primary care challenges, reflecting key difficulties for our care partners not measured by our quantitative scales. While one-third of the sample mentioned cognitive decline as a secondary challenge, it was usually in conjunction with another more primary challenge as care partners often attributed BPSD or functional issues to the PLwD’s declining cognition.

The most common IADLs/ADLs that care partners reported assisting with were medication management (86%), transportation (84%) and shopping (84%). The most distressing tasks, including assisting with toileting, cleaning, mobility tasks, and dressing the PLwD from the waist down, were managed by a small proportion of care partners and reflect more basic care tasks associated with greater severity of dementia. The most common BPSDs reported included apathy (72%), agitation (71%), and dysphoria (52%), while the most distressing included aggression, agitation, and disinhibition. Agitation (the most common challenge in the qualitative analysis) was the second most distressing BPSD (behind the less frequent aggression) and the second most common quantitatively reported BPSD. Finally, the most common cognitive symptoms included memory and ability to make decisions aligning with the qualitative results. Table 3 portrays the frequency of care partners who encountered each challenge, compares DMS subscale scores (criticism, active management, encouragement) by each specific care challenge (ADL/IADLs, BPSD, and cognitive decline) among those care partners who did and did not report the challenge, and reports associations between the total scale scores for the care challenges and each dementia management strategy.

### 3.2. ADLs/IADLs: Qualitative

Among those who identified ADLs as a primary challenge, ADLs were most frequently managed in a straightforward way (n = 15) with the care partner providing “direct” instructions or in their own words “just doing it”. They took care of the task without giving it much thought by going through the physical motions of assisting with bathing or dressing. For example, participant 145 describes a toileting challenge this way, “I just glove up and I do what has to be done.” Participants engaged with the impaired functioning of the PLwD by “shaping the lived experience” of the PLwD (n = 12), which included preventative actions whereby the care partner took steps to avoid dealing with the same scenario in the future and environmental modifications whereby the care partner changed the environment to better enable the PLwD to complete the task appropriately. For example, participant 196 describes his approach to managing self-care this way, “We got a little game, every night at bedtime: I say ‘time to brush our teeth’. She drags on a little bit… and we brush together… she does it and I help her, then I brush my own teeth, and that’s acceptable to her, because I’m doing it, too.” Here, the care partner is “shaping the lived experience” of the PLwD by modifying his own actions to change how his wife perceives the functional task. No participant reported an IADL as a primary care challenge; thus, care management for these tasks was not described.

### 3.3. ADLs/IADLs: Quantitative

A similar pattern was seen in the quantitative analyses, with active management being the only dementia management strategy significantly correlated with the overall CAFU score (r = 0.35, *p* < 0.01). Care partners who helped with finances (t = −2.03, *p* < 0.05), food preparation (t = −2.45, *p* < 0.05), eating meals (t = −2.45, *p* < 0.05), laundry (t = −2.12, *p* < 0.05), phone calls (t = −2.10, *p* < 0.05), dressing from the waist up (t = −4.05, *p* < 0.001) and waist down (t = −3.13, *p* < 0.01), grooming (t = −2.78, *p* < 0.01), bathing (t = −2.72, *p* < 0.01), and toileting (t = −2.03, *p* < 0.05) had significantly higher active management scores than care partners who did not help with those tasks. Care partners who helped with finances also had higher encouragement scores relative to those who did not help with that IADL (t = −2.10, *p* < 0.05). Criticism was not associated with help with IADLs and ADLs.

### 3.4. Behaviors: Qualitative

In contrast with ADLs, those who listed behaviors as a challenge were more likely to be managed with approaches showing lack of adjustment (n = 14) such as remaining “hands-off”, being “rigid” in approach, appearing “stuck” without knowing how to manage, or “withdrawing” from the situation and PLwD altogether. Participant 107 showed his rigidity through his unwillingness to change his approach: “Sometimes if I say ‘no, no that didn’t happen’—‘yes it did’ and so now I’m conflicted on—like everybody’s told me ‘don’t argue, just go on to whatever’ and again, I go back to my part that you know I just don’t like letting it go.” Care partners addressing behavioral challenges commonly responded with criticism (n = 11), akin to the DMS subscale, by arguing, criticizing, expressing irritation and frustration, or pleading with the PLwD. For example, participant 167′s mother was perseverating on her mail being checked, and the care partner expressed her irritation saying, “Mom, you’ve asked me that 10 times now and I’m getting frustrated with answering 10 times!” Finally, they engaged with the altered behavioral reality of the PLwD (n = 9) in contrasting ways, by either entering the PLwDs lived experience or attempting to pull them back into the care partner’s own reality. Participant 113 entered her mother’s lived experience of terrible nightmares and described her approach as, “physical touch, just to sort of reorient her, reaffirm that you know it’s okay, it’s—you’re safe, the house is not burning down”. This contrasts with participant 152 who attempted to pull her husband back into a normal pattern of behavior and to have him stop following the cat, “My technique is awful, I don’t know what to do, I yell “Joe, Joe, stop it!” or I try to wave my arm in front of him. I don’t know how to divert him…and I’ve tried logic, like that he’s scaring [the cat].”

### 3.5. Behavior: Quantitative

Criticism was the only management strategy significantly associated with overall NPI score (r = 0.34, *p* < 0.01). Likewise, care partners whose care recipient had symptoms of irritability (t = −2.37, *p* < 0.05), hallucinations (t = −2.30, *p* < 0.05), and aggression (t = −3.98, *p* < 0.001) used more criticism than care partners who were not providing care for a PLwD with those symptoms. Care partners who cared for a PLwD with hallucinations also had significantly higher active management scores (t = −2.35, *p* < 0.05); however, encouragement was not associated with BPSD.

### 3.6. Agitation: Qualitative

Like general behavioral management, agitation was most commonly addressed with expressions of criticism (n = 17) or engaging with the different lived experience of the PLwD (n = 11) (specifically by shaping the environment or pulling the PLwD back into the care partners’ lived experience). Participant 166 was dealing with her husband’s agitation as he resisted getting in the car for a trip. She described “So I pleaded for an hour trying to get him to go. I finally reached the threatening stage where I said I was going to call the Sheriff and said he had two choices, either to have the sheriff take him to the hospital or to go with me.” However, another approach toward agitation was the care partner’s attempt to explain (n = 9) through reasoning or giving verbal and visual instructions to achieve a desired end. For example, care partner 151’s mother expressed agitation and resistance to care regarding a trip to the doctor’s office. Care partner 151 stated, “Mom you’ve been in the hospital 4 times already in the last 2 years, you really need to think about that this might be a good thing to do”.

### 3.7. Agitation: Quantitative

As with qualitative reports, greater criticism was reported among care partners of a PLwD with agitation than care partners for a PLwD without agitation (t = −3.44, *p* < 0.001). Agitation was not associated with encouragement or active management.

### 3.8. Cognitive Decline: Qualitative

The management approach for those who mentioned cognitive care challenges was similar to functional care challenges. The most common pattern of response was the straightforward and direct task completion or the “just do it” approach (n = 16). Care partners also engaged with the new cognitive reality of the PLwD by attempting to shape the lived experience through preventative actions or environmental modifications (n = 13). For example, participant 101 modified her actions to help her grandmother perceive a situation differently. Her grandmother would forget and think each day was her hair appointment, but the granddaughter shaped the lived experience by, “Pretending that we did her hair… just like blowing some like hot air on her hair, she falls for that one almost all the time.” However, like the agitation approach, they also used reason or a “tell and show” approach to explain something to the PLwD in attempt to compensate for the cognitive decline (n = 9). For example, as one PLwD would forget the days’ activities, participant 122 described, “every day I would have…a legal pad and I’d say today, here’s what we’re doing.” In a direct way that involved verbal and visual cues, she helped orient her husband to the day’s routine.

### 3.9. Cognitive Decline: Quantitative

Higher levels of cognitive impairment were significantly correlated with active management (r = 0.30, *p* < 0.01). Likewise, care partners whose PLwD was above the mid-severity level on cognitive symptoms of ability to make decisions (t = −3.50, *p* < 0.001), speech and language impairment (t = −2.99, *p* < 0.01), orientation to place (t = −2.11, *p* < 0.05), and recognition of family members (t = −2.31, *p* < 0.05) reported significantly higher levels of active management than care partners whose PLwD was below the mid-severity level. Orientation to place impairment was also associated with significantly less use of encouragement (t = 2.02, *p* < 0.05). Cognitive symptoms were not associated with criticism as a care management strategy.

## 4. Discussion

Utilizing a convergent parallel mixed-methods design [17], this study suggests clear patterns of common distressing symptoms of dementia and how they are managed, with the qualitative data providing nuance about what behavioral management looks like in response to these symptoms. In this study, we take a cross-sectional snapshot of how care partners are managing specific challenges; hence, we view care management as situationally driven. From our quantitative analyses, active management strategies appeared to be a reflection of greater functional and cognitive impairment associated with greater disease severity and required supervision by the care partner. On the other hand, aligning with prior research, criticism was associated with severe behavioral disturbances such as agitation and aggression, leading to the highest care partner stress ratings [32]. This may indicate that criticism is a stress response when care partners perceive the situation to be beyond their control and are no longer able to adaptively manage [7,8]. In addition, this response may extend beyond the situational context to the broader caregiver burden and cognitive load the care partner is experiencing [7,33]. In this vein, prior research has found that care partners perceive symptoms to be more stressful if they felt the PLwD had volition over the behavior, which may be how some BPSDs are perceived by care partners (e.g., “they are doing this on purpose”) [34]. Because criticism is the management strategy associated with the most negative outcomes for the care dyad (burden, potentially harmful behavior), helping care partners manage and adapt to particularly stressful BPSDs may be especially critical in care interventions [12]. Encouragement, however, had limited association with any care challenges and may be something that care partners do in spite of any symptoms, or may relate more to other contextual factors such as personality or relation to the PLwD.

While the qualitative findings largely aligned with the quantitative results, they add important insight to understanding how these strategies are applied. For example, in addressing ADLs and cognitive decline, straightforward “active management” strategies dominated care approaches, but more granular strategies emerged such as care partners modifying their own actions to assist with an ADL (e.g., using teeth brushing as a game the dyad did together). Additionally, care partners often used verbal and visual cues to remind the PLwD of something (e.g., the care partner who told her mother how to use toilet paper and took her in the bathroom to show her). As demonstrated by the quantitative results, BPSDs were often managed with critical behaviors, but qualitative findings also showed that care partners were sometimes rigid or disengaged in their responses—withdrawing rather than criticizing. Yet, even with a challenging behavior such as hallucinations, some care partners found creative ways to engage with the PLwD in their altered mental status (e.g., talking to the hallucination and asking it to leave). It is also important to acknowledge that secondary challenges (i.e., challenges that were listed without a stated care management response) were reported in care partners’ descriptions almost as frequently as primary challenges. In particular, cognitive decline was often mentioned in conjunction with BPSD and/or ADLs. This demonstrates that caregiving challenges are multi-faceted and may require adaptable responses that encompass all facets of the disease (e.g., cognitive, behavioral, functional).

Understanding how care partners use behavioral approaches is critical because they impact key care outcomes for the dyad. As documented in prior work, coping characterized by avoidance, confrontation, or disengagement is associated with increased depression and anxiety in care partners and increased frequency and severity of BPSD in PLwDs, whereas problem-focused (most akin to active management) and acceptance-based coping has been associated with better mental health and fewer depressive symptoms [24,25,34,35,36,37]. Dementia care management strategies, specifically, criticism, anger, and lower levels of encouragement, have been associated with greater care partner burden, a desire to institutionalize the PLwD, and less experienced caregiving gains (e.g., fulfillment) [12,38,39,40,41,42]. Similarly, studies show that care partners who are not sensitive to the level of functioning of the PLwD and who use more punitive management strategies report increased stress and fewer feelings of competence in care [34,36,43].

Person-centered care and other behavioral intervention approaches may be especially beneficial for care partners facing severe BPSD and other care challenges. Person-centered care aims to match each care recipient’s individuality and preferences with provided care. Because unmet needs of a care recipient may result in BPSD, the person-centered care model provides an alternative way to better recognize and respond to needs through interventions that provide meaningful activities and improved relationship quality. Kim and Park [44] find that person-centered care intervention approaches reduced agitation, depression, other BPSD, and improved quality of life. Yet as recent meta-analytic evidence shows a small, albeit significant, effect size of behavioral interventions, future research should consider patterns of how care partners combine cognitive–behavioral care strategies throughout their care provision in attempt to better target and tailor treatments towards multifaceted care styles [16].

### Caveats

This study is not without limitations. First, the relationship between care symptoms such as BPSDs and management strategies may be bidirectional. The presence of certain BPSDs may elicit certain management strategies or management strategies may bring out certain BPSDs. For example, if a care partner is frustrated or impatient, this may increase the PLwD’s agitation. Yet those who provide care for PLwDs who are agitated may also use different management strategies than care partners who are not managing this symptom. In either case, aiding care partners in their understanding and approach to managing these symptoms could have a positive impact on the care dyad. Future longitudinal research could help elucidate the stability of care management approaches over the course of caregiving. A second limitation is that we asked care partners to describe a recent challenge but did not require it to be the most frequent or intense for the care partner. Third, management strategies may impact care partners’ outcomes depending on whether they use them consistently (trait-like) or whether they use them in specific response to a stressor (state-like), and this should be explored in future research [45]. Social desirability bias may have led some care partners to avoid reporting critical behaviors, and overall usage of this strategy was low. Finally, because the data are cross-sectional, the sustainability and effectiveness of changes in approach over time cannot be determined.. Despite the limitations mentioned, this study’s mixed-methods approach to examining care management advances prior work by offering greater depth to understanding how care partners approach care.

## 5. Conclusions

In conclusion, we found that care partners reported BPSD as the most challenging dementia symptoms, particularly agitation which was reported as frequent and consistently stressful across mixed-method assessments. Care partners used various approaches for different care challenges—with consistent patterns across qualitative and quantitative analysis. In particular, BPSD tended to coincide with more critical, rigid, and disengaging management strategies which may reduce well-being for both care partners and PLwDs. The data also demonstrate a variety of strength-based approaches generated by caregivers toward person-centered care that may be fruitful to incorporate in future behavioral interventions. Tailored interventions that target care partners’ specific management styles and their unique care stressors may improve care provision and mental health.

## Figures and Tables

**Table 1:** Sample characteristics.

	Sample (N = 100)
Characteristics	M (SD), Range/%
Age	63.73 (16.15), 20–90
Female	74%
Non White	18%
Married	74%
College degree	72%
Some financial difficulty	20%
Employed	33%
Religious	73%
Relation to CR	
Spouse	59%
Child	31%
Other	10%
CR Dementia Diagnosis	
Alzheimer’s	52%
LBD	16%
Other	32%
Months CG has provided care	55.32 (43.07), 4–220

Note. CR = care recipient, CG = care partner.

**Table 2:** Primary and secondary qualitative care challenges.

Care Challenge	Primary	Secondary
	Frequency, % of Total	Frequency, % of Total
BPSD	51, 44%	16, 21%
Agitation	25	9
Delusions	6	1
Perseveration	5	1
Other	15	5
Cognitive decline	22, 19%	26, 33%
Executive dysfunction	10	11
Memory	9	14
Other	3	1
ADL (ex. eating)	17, 15%	16, 21%
IADL (ex. shopping)	0	6, 8%
Care coordination	11, 10%	5, 6%
Medical care (ex. fall)	7, 6%	5, 6%
Struggle with decline/autonomy	6, 5%	4, 5%
No challenge	1	--
Total care challenges	115	78

Note. BPSD = Behavioral and psychological symptoms of dementia, ADL = Activities of daily living, IADL = Instrumental activities of daily living. Only specific symptoms with a frequency of 5 or greater are listed under their category heading.

**Table 3:** Activities of daily living, neuropsychiatric symptoms, and cognitive symptoms by frequency in the sample and associated distress.

IADLs/ADLs	% of CGs Who Help with Task	Mean Distress Score for Task (SD)	Criticism *t* (df)	Active Management *t* (df)	Encouragement *t* (df)
Medication	86%	0.45 (0.821)	0.45 (98)	−1.56 (98)	−0.28 (98)
Travel	84%	0.57 (0.935)	−0.68 (98)	−1.25 (98)	1.66 (98)
Shopping	84%	0.58 (0.857)	−0.17 (98)	−1.41 (98)	1.51 (98)
Finances	82%	0.57 (1.031)	−0.88 (98)	−2.03 (98) *	−2.10 (98) *
Food preparation	72%	0.58 (0.750)	1.34 (98)	−2.45 (98) *	0.81 (98)
Laundry	71%	0.33 (0.631)	−1.65 (98)	−2.12 (98) *	0.92 (98)
Cleaning	70%	0.86 (0.967)	−1.46 (98)	−1.79 (98)	0.34 (98)
Phone	65%	0.55 (0.902)	−0.71 (98)	−2.10 (98) *	−1.02 (98)
Dressing waist up	54%	0.54 (0.794)	−1.03 (98)	−4.05 (98) ***	0.71 (98)
Grooming	52%	0.65 (1.008)	−1.24 (98)	−2.78 (98) **	0.61 (98)
Bathing	49%	0.76 (1.011)	−1.47 (98)	−2.72 (98) **	−0.12 (98)
Dressing waist down	48%	0.77 (0.857)	−0.88 (98)	−3.13 (98) **	1.14 (98)
Toileting	32%	1.09 (1.088)	−0.177 (98)	−2.03 (98) *	1.86 (98)
Mobility	31%	0.77 (0.920)	−0.92 (98)	−1.75 (98)	0.53 (98)
Eating meals	18%	0.50 (0.618)	−0.98 (98)	−2.45 (98) *	1.25 (98)
CAFU sum (*r*)			0.10	0.35 **	−0.15
**Neuropsychiatric Symptoms**	**Frequency of occurrence in sample (%)**	**Mean distress score (SD)**	**Criticism** ***t* (df)**	**Active** **Management** ***t* (df)**	**Encouragement** ***t* (df)**
Apathy	72%	1.96 (1.283)	−0.67 (98)	−1.26 (98)	−0.24 (98)
Agitation	71%	2.70 (1.040)	−3.44 (98) ***	−0.78 (98)	−0.89 (98)
Dysphoria	52%	2.29 (1.194)	−1.08 (97)	1.00 (97)	−1.01 (97)
Appetite and eating disorders	47%	1.83 (1.340)	−0.89 (97)	−0.63 (97)	−0.78 (97)
Anxiety	46%	2.54 (1.260)	−1.89 (98)	−0.90 (98)	−0.82 (98)
Irritability	45%	2.09 (1.184)	−2.37 (98) *	0.20 (98)	−0.71 (98)
Motor disturbance	42%	1.93 (1.421)	−0.80 (98)	−1.42 (98)	1.09 (98)
Disinhibition	38%	2.61 (1.424)	−1.92 (98)	−0.63 (98)	0.10 (98)
Sleep disorders	34%	2.06 (1.560)	0.48 (98)	0.53 (98)	1.75 (98)
Delusions	34%	2.53 (1.080)	−0.36 (98)	−0.85 (98)	−0.85 (98)
Hallucinations	28%	1.79 (1.424)	−2.30 (98) *	−2.35 (98) *	−0.54 (98)
Aggression	22%	2.86 (0.990)	−3.98 (98) ***	−0.70 (98)	−0.03 (98)
Aberrant vocalizations	17%	1.88 (1.364)	−0.68 (98)	−1.48 (98)	1.57 (98)
Euphoria	8%	0.75 (0.886)	−0.62 (98)	0.14 (98)	0.95 (98)
NPI-C (*r*)			0.34 **	0.19	0.05
**Cognitive Symptoms**	**% above mid-severity level**	**Impairment Score Mean (SD)**	**Criticism** ***t* (df)**	**Active** **Management** ***t* (df)**	**Encouragement** ***t* (df)**
Memory (Range: 0–6)	64%	4.05 (1.266)	−1.17 (98)	−0.71 (98)	1.36 (98)
Ability to Make Decisions (Range: 0–4)	56%	2.56 (1.076)	−0.88 (98)	−3.50 (98) ***	0.50 (98)
Orientation to Time (Range: 0–4)	33%	1.83 (1.240)	−0.53 (98)	−1.94 (98)	0.93 (98)
Speech and language (Range: 0–6)	29.3%	2.60 (1.538)	−0.58 (97)	−2.99 (97) **	0.61 (97)
Orientation to Place (Range: 0–4)	25%	1.77 (1.213)	−0.22 (98)	−2.11 (98) *	2.02 (98) *
Recognition of Family Members (Range: 0–5)	20%	1.28 (1.408)	0.04 (98)	−2.31 (98) *	1.29 (98)
DSRS Cognitive item sum (*r*)			0.06	0.30 **	−0.17

Note. CG = care partner; Bolded rows indicate section headers for different symptom categories. The top three mean distress scores on the CAFU and NPI scales are bolded to draw attention to the most distressing symptoms for care partners. NPI distress is on a scale from 0—not distressing to 5—extremely distressing, and CAFU distress is on a scale from 0—not at all stressful to 4—extremely stressful. *** *p* < 0.001, ** *p* < 0.01, * *p* < 0.05.

## Data Availability

The data presented in this study are available on request from the corresponding author due to privacy and ethical concerns.

## Supplementary Materials

The following supporting information can be downloaded at: https://www.mdpi.com/article/10.3390/ijerph23020254/s1, Table S1: Enacted care strategies, code definitions, and participant quotes.
