# Supplementary material for: “I Just Glove up and Do What Has to Be Done”: A Mixed-Methods Exploration of Dementia Care Challenges and Care Management Strategies
Source: Int J Environ Res Public Health. 2026 Feb 17;23(2):254. doi: 10.3390/ijerph23020254 (PMC12940411; doi:10.3390/ijerph23020254)
Supplement: Supplementary file 1 [file ijerph-23-00254-s001.zip › ijerph-4115445-supplementary.pdf]

Supplementary Table S1. Enacted Care Strategies, Code Definitions, and Participant Quotes

**Code**	**Definition**	**Exemplar quote**
Anger	Expressing anger, irritation, frustration, arguing or criticizing	ID 141: "I end up getting irritated at her when she just won't accept I'm telling her something that's factual and she thinks – I don't know what she thinks. She doesn't acknowledge the information."<br>ID 102: "ultimately losing your temper and saying "[explative], why don't you get a hearing aid" and saying it very loud and so forth…"
Direct	a straightforward, linear or directive approach	ID 122: "every day I would have… a legal pad and I'd say today, here's what we're doing."<br>ID 178: "I simply remind her of what needs to be done and for what reasons…"
Enter lived experience	recognizing CR in different reality; changing CG behavior or thought process to accommodate CR reality	ID 157: "…like, there's two of me. It's gotten to the point now, it's just our normal. He asks me about where his wife is, 'I haven't seen her but if I do, I'll let you know'."<br>ID 159: "He, at night, will wake up and there will be a man in a black coat, 'they're taking the house away from us…', so then what I've done is I have to get up, I come out here and I open the door wall and I yell and scream at these men - they have to get out, they're not allowed to come back."
Environmental modifications	modifying the lived-in environment (e.g., unscrewing lightbulbs, sticky notes on mirror)	ID 139: "I have to help her get into the shower – we have a regular tub – and I put handrails, safety rails on it so she can hold on, and I can help her in."<br>ID 148: "My mother has gotten so she pees in the trash can, so I can't keep the trash can in the bathroom anymore."
Hands off	Passive or removed management	ID 100: "I usually don't argue with him anymore and then I just try to watch out for him."<br>ID 104: "Just leave her alone really."
Just do it	'In-vivo' code of participants describing their independent management of a task	ID 145: "I just glove up and I do what has to be done."<br>ID 190: "Just go there and do it."
Preventative actions	taking an action in effort to prevent a future negative outcome	ID 131: "remind her that the toilet paper goes in the toilet. And so… when I know she's gonna go to the bathroom, or I ask her to go to the bathroom, I then remind her – toilet paper goes in the toilet."<br>ID 162: "during the day I can remind him to change, and he wears Depends so that's pretty well managed during the day and so at night, what I do is put a tarp on the sheet under where he is and then I put a waterproof pad over that so that it's softer for him to lie on."
Reason	trying to use logic or explain something in a rational way to CR	ID 151: "I said 'mom you've been in the hospital 4 times already in the last 2 years, you really need to think about that this might be a good thing to do.'"

		ID 191: "Finally, I have to kind of talk to her like a kid – 'would I lie to you? Do you not believe that I'm doing this because I love you? – Well, I know you do – Then why don't you just let me do what I know I need to do?'"
Rigidity	absence of change in management strategy or adjustment of perspective	ID 107: "like everybody's told me 'don't argue, just go on to whatever' and again, I go back to my part that you know I just don't like you know just letting it go."<br>ID 126: "So, last week…he went out and sat in the car, in the passenger seat and put on a seatbelt and I went out and asked him why he was there and he couldn't respond and I asked him to come to bed and he didn't respond so he sat there for an hour and a half in the dark with the seatbelt on… so I decided I would go to bed. So I did."
Shape lived experience	modify CG reality to alter landscape of CR reality	ID 101: "Pretending that we did her hair, that works really well too – just like blowing some like hot air on her hair, she falls for that one almost all the time."<br>ID 196: "We got a little game, every night at bed time I say time to brush our teeth. She drags on a little bit… and we brush together… she does it and I help her, then I brush my own teeth, and that's acceptable to her, because I'm doing it, too."
Stuck	Floundering, not knowing how to respond	ID 155: "How do I respond to either have him not ask again, which I haven't figured that out, or just how I deal with it. Right now, it's just repeating the answer because I haven't figured out how"<br>ID 166: "Fortunately his friend was home and came over and talked very sternly with my husband…I honestly don't know what I would have done to get him to go."
Tell and show	Using both verbal and visual cues	ID 131: "sometimes I have mom come into the bathroom even though I know she's not gonna remember, and show her, just trying to prompt that little bit of memory there, to get her to, and remind her that the toilet paper goes in the toilet."<br>ID 171: "there's no sense in telling him 'be sure to use the microwave'. He's at a stage where the microwave, he maybe can't find it, showing him is better than verbal – he's having trouble with verbal instruction."
Withdraw	physical removing of self from CR e.g., walking into another room	ID 157: "But those things that get tense, I have to shut my mouth and walk away or I might say something nasty."<br>ID 147: "I walk away – I take a break for a little bit because I'm getting too frustrated and I think about it, it's not her fault."
